# Antidepressants normalize elevated Toll-like receptor profile in major depressive disorder

**DOI:** 10.1007/s00213-015-4087-7

**Published:** 2015-09-29

**Authors:** Yi-Yung Hung, Kai-Wei Huang, Hong-Yo Kang, Gloria Ya-Ling Huang, Tiao-Lai Huang

**Affiliations:** Department of Psychiatry, Kaohsiung Chang Gung Memorial Hospital and Chang Gung University College of Medicine, 123, Ta-Pei Rd, Niao-Sung, Kaohsiung, 833 Taiwan Republic of China; Graduate Institute of Clinical Medical Sciences, Chang Gung University, College of Medicine, Kaohsiung, Taiwan; Center for Menopause and Reproductive Research, Kaohsiung Chang Gung Memorial Hospital, Kaohsiung, Taiwan; Department of Nursing, Kaohsiung Chang Gung Memorial Hospital and Chang Gung University College of Medicine, Kaohsiung, Taiwan; Genomic and Proteomic Core Laboratory, Department of Medical Research, Kaohsiung Chang Gung Memorial Hospital, Kaohsiung, Taiwan

**Keywords:** Toll-like receptor, Innate immunity, Major depressive disorder, Inflammation

## Abstract

**Rationale:**

Abnormalities in Toll-like receptor (TLR) expression in depression have been inferred in part from observed increases in TLR4 levels in peripheral blood mononuclear cells (PBMCs) and postmortem brains of depressed and suicidal patients. Our previous study found differences in the TLR expression in PBMCs between healthy controls and patients with major depressive disorder. Normalization of increased TLR4 in PBMCs by cognitive behavior psychotherapy has been reported. However, the effects of antidepressants remain unknown.

**Objectives:**

Changes in TLR1–9 expression levels of PBMCs were examined in 56 patients with MDD. The 17-item Hamilton Depression Rating Scale (HAMD-17) and mRNA expression levels of TLRs were assessed in parallel with a housekeeping gene using qRT-PCR before and after treatment with antidepressants.

**Results:**

TLR3, TLR4, TLR5, TLR7, TLR8, and TLR9 were expressed at elevated levels in patients with MDD and were significantly decreased by treatment with antidepressants for 4 weeks. Antidepressant treatment completely normalized TLR3, TLR5, TLR7, TLR8, and TLR9 levels, whereas TLR1, TLR2, TLR4, and TLR6 were decreased to below normal levels. A subgroup analysis found that only TLR3 was significantly higher at baseline in the nonremission group. In addition, a multiple linear regression analysis revealed that only low TLR3 before treatment predicted improvement in HAMD-17 scores.

**Conclusions:**

These findings suggest that antidepressant treatment exerts anti-inflammatory effects in patients with MDD and identify TLR profiles as a predictor of response to antidepressant therapy. Further studies investigating the effects of manipulating individual TLRs on depression are needed to fully elucidate the underlying mechanism.

## Introduction

Major depressive disorder (MDD) represents a combination of mood, cognition, sleep, and appetite disturbances that lasts for at least 2 weeks. MDD accounts for 7.4 % of total disability-adjusted life years worldwide (Whiteford et al. 2013). MDD is thus consistently a leading source of disease burden worldwide (Lopez et al. 2006) MDD often negatively affects the people around the patient, and the burden extends to family, coworkers, employers, and others (Birnbaum et al. 2010; Lepine and Briley 2011). In addition, depressive disorders are comorbid with other inflammatory diseases, including diabetes, cancer, cardiac disease, and metabolic syndrome (Evans et al. 2005). The inflammatory burden of individuals with depression was highlighted recently (Rethorst et al. 2014).

The etiology of MDD is complex and its pathophysiological association with inflammation remains ill defined. The link between altered immune response and MDD has grown out of the observation that cytokine therapy for different diseases, especially treatment with interferon-α, is accompanied by depressive symptomatology (Capuron and Miller 2004; Miyaoka et al. 1999). About one third of patients who receive recombinant human cytokines for the treatment of hepatitis C exhibit complicating depressive symptoms. Moreover, numerous additional proinflammatory cytokines in peripheral blood, including tumor necrosis factor (TNF)-α, interleukin (IL)-1β, IL-6, and IL-8, have been reported to be elevated in different stage of MDD (Dowlati et al. 2010). We have also found significantly higher serum IL-1β levels and IL-1β/IL-10 ratios in patients with melancholic features compared with patients lacking these features (Huang and Lee 2007).

Extensive evidence supports the importance of proinflammatory cytokines/chemokines in major depression (Mina et al. 2014; Muller 2014). However, given that PRRs (pattern-recognition receptors), especially Toll-like receptors (TLRs) (Table 7), are important modulators of immune homeostasis in peripheral blood, and that signal transduction mediated by these receptors converges on nuclear factor kappa-light-chain-enhancer of activated B cells (NF-κB) and activator protein 1 (AP-1), which lead to the production of proinflammatory cytokines/chemokines, it is likely that deficits in expression are more important than proinflammatory cytokines/chemokines per se in triggering MDD.

Few studies have directly examined alterations in TLR expression in the context of depression. Preclinical studies have shown that TLR4 expression in the prefrontal cortex is enhanced in a stress-based model of depression (Garate et al. 2014; Garate et al. 2013). In addition to changes in central nerve system expression, altered peripheral expression of TLRs appears to be associated with depression and may account for the heightened inflammatory state (Liu et al. 2014). In addition, some antidepressant and anti-inflammatory agents are able to attenuate the depressive-like behavior induced by lipopolysaccharide, a TLR4 agonist (Habib et al. 2015; Salazar et al. 2012). Postmortem studies have shown enhanced expression of TLR3 and TLR4 mRNA and protein in the dorsolateral prefrontal cortex of depressed suicide victims (Pandey et al. 2014). Moreover, clinical studies have reported upregulated TLR4 signaling in peripheral blood mononuclear cells (PBMCs) in newly diagnosed patients with MDD, a finding that may be related to leakage of various damage-associated molecular patterns, such as 16S rDNA, through the gut (Keri et al. 2014). In a similar vein, our recent investigation of the expression of TLRs in PBMCs in the acute phase of MDD revealed higher expression of TLR3, TLR4, TLR5, and TLR7, and lower expression of TLR1 and TLR6 in depressed patients. Furthermore, TLR4 expression was found to be an independent risk factor for MDD severity (Hung et al. 2014).

Data on the effects of antidepressants on TLR signaling are limited. In a cell line study, tricyclic antidepressants such as amitriptyline were shown to significantly inhibit TLR2 and 4 signalings (Hutchinson et al. 2010). Selective serotonin reuptake inhibitors (SSRIs), such as fluoxetine and citalopram treatment, have also been shown to inhibit the signaling of TLR3, TLR7, TLR8, and TLR9 in mice with collagen-induced arthritis (Sacre et al. 2010).

Apart from these reports, few studies have extensively investigated the effects of antidepressants on the expression of TLRs and their association with clinical outcome. Accordingly, the present study was designed to monitor changes in the expression of TLRs before and after treatment with antidepressants and investigate their association with clinical outcome.

## Material and methods

### Experimental design

The study population (*n* = 56) was recruited from inpatients with ongoing MDD in the psychiatric ward of Kaohsiung Chang Gung Memorial Hospital Medical Center, Taiwan, from August 2013 to May 2015. Blood samples for TLR mRNA analysis were obtained from patients and 35 healthy controls at baseline. Some pretreatment data were published in our previous work (Hung et al. 2014). Only patients who received follow-up and therapy as usual for 4 weeks were included. Institutional Review Board approval was obtained from the hospital ethics committee (101-5012A3 and 103-5114B). Patients and healthy controls provided written consent to participate after receiving verbal and written information about the study.

### Participants

Patients with major depression were screened by two psychiatrists before entering the study. The screening involved three steps: (1) a Structured Clinical Interview for DSM-IV Axis I Disorders, (2) a detailed assessment of previous psychiatric symptoms and treatment, and (3) an assessment of severity of depression using the 17-item Hamilton Depression Rating Scale (HAMD-17) (Hamilton 1960) performed by the same psychiatrist. Patients with psychotic disorder, alcohol dependence, severe metabolic syndrome, a body mass index (BMI) > 34 kg/m^2^, a history of any inflammatory disease, or who were taking anti-inflammatory or immune modulating drugs were excluded from the study. All patients were also tested for blood pressure and received chest X-rays, electrocardiogram examinations, and routine blood tests after hospitalization to exclude any chronic physical illness, including heart, lung, liver, kidney, or uncontrolled metabolic diseases. Patients were also checked for acute infections or allergic reactions. None reported taking any antidepressants for at least 1 week before entering the study. Healthy controls, recruited from the community, received a similar clinical evaluation and had neither a personal history nor a first-degree relative with psychiatric disorder. The same psychiatrist who performed screens of MDD patients assessed the healthy control group using Chinese Health Questionnaire-12 and Diagnostic and Statistical Manual of Mental Disorders (fourth edition) criteria to rule out psychiatric disease. Table 1 shows the demographic findings and clinical data for healthy controls and MDD patients.

**Table 1:** Demographic findings and clinical data for healthy controls and MDD patients

	Major depression (*n* = 56)	Healthy controls (*n* = 35)	*p* value
Age (years)	45.62 ± 10.43	39.80 ± 12.00	0.017*
Gender (M/F)	18/38	10/25	0.723
BMI (kg/m^2^)	23.45 ± 4.81	22.64 ± 2.78	0.314
Education duration (years)	11.38 ± 3.09	15.74 ± 3.06	0.000*
Smoking	13	3	0.052
HAMD-17	27.83 ± 4.97	–	–

*Independent *t* test, *p* < 0.05

After clinical examination, blood samples were taken at two time points: at inclusion (baseline) and 4 weeks after initiating antidepressant treatment. Between these two time points, the patients were hospitalized in the same ward with good drug adherence, regular sleep-wake cycles, a well-controlled diet, and limited smoking.

Of the 56 patients initially enrolled, 43 completed the longitudinal part of the study. The reasons for drop out included changing antidepressants owing to intolerable side effects (*n* = 7) and refusing follow-up assessment (*n* = 6). Dropouts did not differ from those completing the study with regard to baseline TLR mRNA levels or clinical characteristics.

### Treatment

Treatment was given as dictated by medical considerations, meaning that the choice of treatment was not influenced by the study and was chosen based on clinical judgment. After screening at baseline, chosen antidepressants were administered and recorded. The antidepressants included escitalopram (10–20 mg/day; *n* = 11), fluoxetine (40–80 mg/day; *n* = 9), paroxetine (20–40 mg/day; *n* = 8), duloxetine (60–120 mg/day; *n* = 4), venlafaxine (37.5–225 mg/day; *n* = 5), bupropion (150–300 mg/day; *n* = 2), mirtazapine (45 mg/day; *n* = 1), and agomelatine (25–50 mg/day; *n* = 2). All patients received benzodiazepines as anxiolytics or hypnotics. Supportive psychotherapy sessions were provided one to two times, and regular activities were suggested during hospitalization.

### Quantitative reverse transcription-polymerase chain reaction (qRT-PCR) analysis

Venous blood (5 mL) samples were drawn into PAXgene Blood RNA Tubes between 8:00 am and 10:00 am, after the patients had fasted for 9 h. The tubes were placed in a -80 °C freezer immediately after collection and were stored there until they were assayed. The protocol used for TLR mRNA analyses was the same as that used in our previous study (Hung et al. 2014). The relative abundance of TLR mRNAs was assessed by the comparative Ct method using glyceraldehyde-3-phosphate dehydrogenase (GADPH) as a housekeeping gene.

### Statistical analysis

All results are presented as means ± standard deviation. Chi-squared was used to compare differences in demographic data (e.g., sex), and Student’s *t* test was used to compare differences in age and BMI. The distribution of TLR1 and TLR2 in healthy controls was skewed. All other TLRs were normally distributed initially (TLR3, TLR6, TLR7, TLR8, TLR9) or after excluding outliers (TLR4, *n* = 7 patients at baseline; TLR5, *n* = 9 healthy controls) based on a boxplot outlier detection rule. The two-tailed Mann–Whitney test was used to compare differences in TLR1 and TLR2 expression levels between patients and controls, and a nonparametric two-paired-sample test was used to compare differences between baseline and follow-up. Independent *t* tests were used to compare differences in TLR4, TLR5, TLR6, and TLR9 expression levels between patients and controls, and paired *t* tests were used to compare differences between baseline and follow-up. Age has been reported as a confounder in TLR3, TLR7, and TLR8 mRNA expression (Panda et al. 2010); thus, an age-adjusted analysis of covariance (ANCOVA) was used to compare differences in the expression of these isoforms. Because sample size was relatively small after dividing the 43 completers into remission and nonremission groups (cutoff ≤ 7), a two-tailed Mann–Whitney test was used to compare differences at baseline. A linear regression model with forward-selection was use to establish the relationship between baseline TLR mRNA expression levels and changes in HAMD-17 after antidepressant treatment for 4 weeks. All statistical analyses were performed using Statistical Product and Service Solutions (SPSS), version 22. For each test, *p* < 0.05 was considered significant.

## Results

### Baseline TLR mRNA levels in patients compared with controls

mRNA levels for six of the nine TLRs were significantly elevated in depressed patients at baseline compared with healthy controls. Independent *t* tests showed significantly elevated expression of TLR4, TLR5, and TLR9 in patients with MDD, whereas age-adjusted ANCOVAs showed significantly higher expression of TLR3, TLR7, and TLR8. No significant differences were found for TLR1, TLR2, or TLR6. Comparisons of baseline expression of TLRs between MDD patients and healthy controls are summarized in Table 2.

**Table 2:** Baseline TLR mRNA expression levels (−delta Ct) in MDD patients and healthy control subjects

	Patients (*n* = 56)	Controls (*n* = 35)	*p* value
TLR1	−1.72 ± 1.69	−1.42 ± 1.26	0.253
TLR2	0.24 ± 3.87	−1.08 ± 1.32	0.060
TLR3	−5.69 ± 3.65	−7.49 ± 1.68	0.026*
TLR4	−0.079 ± 2.90	−1.02 ± 1.30	0.043*
TLR5	−3.27 ± 4.04	−4.56 ± 1.10	0.027*
TLR6	−3.83 ± 3.93	−4.23 ± 1.53	0.499
TLR7	−3.31 ± 2.71	−4.66 ± 1.31	0.032*
TLR8	1.12 ± 3.56	−0.33 ± 0.71	0.039*
TLR9	−5.21 ± 3.76	−6.41 ± 1.10	0.029*

Two-tailed Mann–Whitney test (TLR1 and TLR2); two-tailed independent *t* test (TLR4, TLR5, TLR6, and TLR9); age-adjusted ANCOVA (TLR3, TLR7, and TLR8)

*p < 0.05

### Outcomes after 4 weeks of therapy

Forty-three patients completed the 4 weeks of therapy and were reassessed with the same tools as used at inclusion. HAMD-17 scores for completers were significantly reduced at endpoint (8.47 ± 5.85) compared with baseline (27.91 ± 5.10). For completers, mRNA expression for all TLRs, regardless of elevation status at baseline, was significantly reduced after 4 weeks of therapy compared with baseline. HAMD-17 scores and mRNA abundance for all TLRs at baseline and endpoint are summarized in Table 3.

**Table 3:** TLR1–9 mRNA levels (−delta Ct) of MDD patients at baseline and at week 4

	Baseline (*n* = 56)	After 4 weeks (*n* = 43)	*p* value
HAMD	27.91 ± 5.10	8.47 ± 5.85	0.000**
TLR1	−1.72 ± 1.69	−2.56 ± 2.01	0.015*
TLR2	0.24 ± 3.87	−2.96 ± 5.15	0.000**
TLR3	−5.69 ± 3.65	−8.34 ± 4.78	0.000**
TLR4	0.19 ± 3.83	−2.67 ± 5.14	0.002**
TLR5	−3.27 ± 4.04	−6.41 ± 5.33	0.000**
TLR6	−3.83 ± 3.93	−6.52 ± 4.91	0.000**
TLR7	−2.72 ± 3.62	−5.74 ± 4.87	0.000**
TLR8	1.12 ± 3.56	−1.04 ± 4.61	0.001**
TLR9	−5.21 ± 3.76	−7.10 ± 4.59	0.005**

Wilcoxon signed-rank test (TLR1 and 2); Paired *t* test (TLR3-9)

*p < 0.05; **p < 0.01

TLR3, TLR5, TLr7, TLR8, and TLR9, which showed elevated mRNA levels at baseline (see Table 2), were normalized by antidepressant treatment, as evidenced by the insignificant difference in abundance between patients and controls at week 4, whereas TLR1, TLR2, TLR4, and TLR6 were decreased to levels below baseline controls after antidepressant treatment (Table 4).

**Table 4:** TLR1–9 mRNA levels (−delta Ct) in MDD patients after 4 weeks of treatment compared with baseline values in healthy controls

	After 4 weeks (*n* = 43)	Controls (*n* = 35)	*p* value
TLR1	−2.56 ± 2.01	−1.42 ± 1.26	0.002**
TLR2	−2.96 ± 5.15	−1.08 ± 1.32	0.012*
TLR3	−8.34 ± 4.78	−7.49 ± 1.68	0.432
TLR4	−2.67 ± 5.14	−1.02 ± 1.30	0.049*
TLR5	−6.41 ± 5.33	−4.72 ± 1.42	0.051
TLR6	−6.52 ± 4.91	−4.23 ± 1.53	0.006**
TLR7	−5.74 ± 4.87	−4.66 ± 1.31	0.282
TLR8	−1.04 ± 4.61	−0.33 ± 0.71	0.668
TLR9	−7.10 ± 4.59	−6.60 ± 1.58	0.508

Two-tailed Mann–Whitney test (TLR1 and TLR2); two-tailed independent *t* test (TLR4, TLR5, TLR6, and TLR9); age-adjusted ANCOVA (TLR3, TLR7, and TLR8)

*p < 0.05; **p < 0.01

Of the 43 completers, 24 patients showed HAMD-17 scores < 7, classified as remission, whereas 19 patients did not reach remission criteria. Only TLR3 level was significantly lower in the remission group (−8.34 ± 4.78) than in the nonremission group (−7.49 ± 1.68) (Table 5). Moreover, a linear regression analysis showed that only TLR3 levels were predictive of changes in HAMD-17 scores (Tables 6 and 7).

**Table 5:** Differences in TLR mRNA levels at baseline for patients in remission and nonremission groups

	Remission (*n* = 24)	Nonremission (*n* = 19)	*p* value
TLR1	−2.56 ± 2.01	−1.42 ± 1.26	0.732
TLR2	−2.96 ± 5.15	−1.08 ± 1.32	0.261
TLR3	−8.34 ± 4.78	−7.49 ± 1.68	0.035*
TLR4	−2.67 ± 5.14	−1.02 ± 1.30	0.156
TLR5	−6.41 ± 5.33	−4.72 ± 1.42	0.250
TLR6	−6.52 ± 4.91	−4.23 ± 1.53	0.212
TLR7	−5.74 ± 4.87	−4.66 ± 1.31	0.136
TLR8	−1.04 ± 4.61	−0.33 ± 0.71	0.136
TLR9	−7.10 ± 4.59	−6.60 ± 1.58	0.070

*Two-tail Mann–Whitney test, *p* < 0.05

**Table 6:** Relationship between changes in HAMD-17 score and TLRs at baseline

Independent factors	Change in HAMD-17 score		
	Standardized coefficients	*t*	*p* value
TLR1	0.026	0.137	0.892
TLR2	−0.430	−0.703	0.487
TLR3	−1.226	−2.045	0.049^*^
TLR4	1.317	1.347	0.187
TLR5	−0.123	−0.247	0.806
TLR6	0.686	1.278	0.210
TLR7	0.178	0.197	0.845
TLR8	−0.535	−0.455	0.652
TLR9	−0.127	−0.202	0.841

*Linear regression with forward-selection, *p* < 0.05

**Table 7:** Functions of different TLRs

	Ligands		Cytokine produced
	DAMP	PAMP	
TLR1/TLR2	HSP	Triacyl lipoproteins	TNF-α, IFN-γ, IL-12, TGF-β, IL-10, IL-23
TLR2/TLR6	HMGB1	Peptidoglycan	
TLR3	Self dsRNA	Viral dsRNA	IFN-β
TLR4	HSPs	HSPs	IFN-γ, IL-6, IL-10, IL-12
	Fibrinogen	Lipopolysaccharides	
	Heparan sulfate	RSV fusion protein	
	Fibronectin	MMTV	
	Hyaluronic acid	paclitaxel	
	HMGB1		
TLR5		Flagellin	TNF-α, IL-1, IL-6, IL-8
TLR7	Self ssRNA	Viral ssRNA	IFN-β, IL-12 p70
TLR8	Self ssRNA	Viral ssRNA	IL-6, IL-10, IL-12, and TNF-α
TLR9	Self-DNA	Bacterial and viral DNA	IL-6, IL-10

*TLRs* Toll-like receptors, *DAMP* damage-associated molecular patterns, *PAMP* pathogen-associated molecular pattern, *HSP* heat shock protein, *HMGB1* high-mobility group protein B1, *MMTV* mouse mammary tumor virus

## Discussion

To the best of our knowledge, this is the first study to evaluate changes in TLRs in PBMCs during antidepressant treatment in patients with MDD. Inpatient treatment and follow-up in this study controlled for all possible confounding factors, including diet, smoking and drug compliance. There are four main findings of this study. First, the relative mRNA expression levels of all TLRs in PBMCs surprisingly decreased after treatment with antidepressants (mainly) for 1 month. Second, following treatment, TLR1, TLR2, and TLR6 mRNA levels in PBMCs were lower than those in healthy controls, whereas mRNAs for TLR3, TLR4, TLR5, and TLR7 in PBMCs, were normalized. Third, pretreatment TLR3 mRNA expression levels were significantly higher in the nonremission subgroup than in the remission group. Finally, only elevated TLR3 mRNA level in PBMCs before treatment was associated with a diminished decrease in HAMD score during antidepressant treatment in patients with MDD.

This study showed that mRNA expression levels of all TLRs measured were decreased during antidepressant treatment in patients with MDD. The elevated levels of TLR3, TLR4, TLR5, and TLR7 reported in our previous work (Hung et al. 2014) were normalized by antidepressant treatment. Few studies have investigated interactions among treatment conditions, TLRs, and the clinical course of MDD. In one study on the effects of antidepressants on TLRs in vitro, Hutchinson et al. (2010) reported that amitriptyline decreased TLR2 and 4 signalings (Hutchinson et al. 2010). SSRIs, such as escitalopram and fluoxetine, have also been shown to decrease TLR3, TLR7, TLR8, and TLR9 signalings (Sacre et al. 2010), and chronic treatment with fluoxetine has been shown to significantly decrease expression of the *TLR4* gene (Habib et al. 2015). Despite this, research on the long-term effects of antidepressants on all TLRs in vivo is inconclusive, and the mechanism responsible for the generalized decrease in TLR expression is unknown.

The current study found that only lower pretreatment TLR3 mRNA levels predicted greater improvement in HAMD scores after treatment with antidepressants. Maternal immune activation with the TLR3 agonist poly I:C during mid-pregnancy increases anxiety- and depressive-like behaviors. A genome-wide association study identified TLR3 as genetic risk factor for suicide in individuals with asthma and with a familial risk for asthma (Darlington et al. 2014). A postmortem study identified abnormal TLR3 over-expression in the dorsolateral prefrontal cortex of 22 depressed suicide victims (Pandey et al. 2014). In our previous study, we found that TLR3 mRNA was also elevated in PBMCs of MDD patients (Hung et al. 2014). Despite these previous findings, the current study is still the first to establish that pretreatment TLR3 mRNA level is an important predictor of the response to treatment with antidepressants. The possible mechanism that explains the finding is the inhibitory role for TLR3 on neuronal progenitor cells proliferation and neurogenesis (Barak et al. 2014; Okun et al. 2010).

A secondary subgroup analysis found that the nonremission group showed significantly higher pretreatment TLR3 mRNA levels than the remission group. To our knowledge, no previous studies have ever reported an association between clinical outcome and the expression of TLRs. Activation of TLRs would cause excessive production of proinflammatory cytokines. Higher levels of proinflammatory cytokines, especially IL-6, have been shown to cause hypothalamic–pituitary–adrenal (HPA) hyperactivity, reduce 5-HT levels through activation of the tryptophan-metabolizing enzyme indoleamine-2,3-dioxygenase (IDO) and predict poorer clinical outcome in MDD patients treated with antidepressants (Lanquillon et al. 2000; Schiepers et al. 2005). Despite the relatively small size of groups in this study, our findings strengthen the concept that TLR expression levels are associated with the underlying cause of major depression. Further longitudinal studies with larger samples are needed to confirm whether TLRs levels are related to remission and not to the treatment per se.

Our previous study showed that TLR1 and TLR6 mRNA in MDD patients was significantly lower than that in healthy controls. In the current work, these two TLRs, in addition to TLR2, were the only forms that decreased significantly after antidepressant treatment. Interestingly, TLR2, which acts in concert with TLR1 or TLR6 as a heterodimer (Jin et al. 2007; Triantafilou et al. 2006), is essential for recognizing bacterial lipoproteins and lipopeptides (Takeuchi et al. 2001; Takeuchi et al. 2002). TLR2 is pivotal for both homeostasis and immune surveillance of the CNS (Rivest 2009). Reported associations of TLR2 genetic variants with Alzheimer’s disease (Yu et al. 2011), onset age of bipolar disorder (Oliveira et al. 2015), and cognitive function in schizophrenia (Kang et al. 2013) are highly suggestive of genetically driven interindividual ability to modulate neuroinflammatory processes. Administration of OxPAPC, a novel TLR2 and TLR4 antagonist, into the CNS prior to stress has been shown to prevent the stress-induced potentiation of hippocampal proinflammatory responses (Weber et al. 2013). TLR1 was proposed to be among the factors that affect the clinical phenotype of schizophrenia and bipolar disorder (de Baumont et al. 2015). However, its role in MDD is still unknown.

### Limitations

One possible limitation of the current study is that the interpretation of results might be influenced by the use of peripheral blood, which may not be affected by antidepressants in the same way as neurons or microglia. Further investigation of the effects of antidepressant on TLRs in neurons is needed to confirm our previous findings. The absence of a suitable healthy control group with the same environmental exposure is another potential limitation. Hospitalization of all patients in the same ward may limit exposure to environmental stimuli. Accordingly, the well-controlled diet, sleep-wake cycle, and alcohol and smoking habits, which are reported to be associated expression of TLRs (Chuffa et al. 2015; Corrigan et al. 2015; Metcalfe et al. 2014; Strekalova et al. 2015), could contribute to differences between patients and healthy controls. Moreover, the remission rate in this study was high compared with double-blind placebo-controlled randomized trials (Gibbons et al. 2012), possibly because of our control over the confounding factors mentioned above.

The effects of different antidepressants may not be the same. The majority of antidepressants used in this study are SSRIs, which have been reported to produce generalized decreases in inflammation, especially in relation to cytokine production. However, imipramine does not follow a similar pattern (Habib et al. 2015) and it has been shown that cytokines themselves can alter TLR expression (Hermoso et al. 2004; Sakai et al. 2004). Elucidating the interactions among antidepressants, cytokines, and TLR expression will require further controlled studies. Finally, the sample size of current study is relatively small and treatment duration may not be long enough. Further analyses using larger sample sizes and longer treatment duration are needed to confirm these results.

## Conclusions

This study has provided novel insights into relationships among TLR expression, antidepressants, and clinical response for a well-characterized MDD. Beyond confirming that the expression levels of certain TLRs are decreased in PBMCs among MDD patients, we have also described—to the best of our knowledge for the first time—distinctive effects of antidepressants on TLRs. In summary, the observed decrease in TLR expression and its association with treatment outcome in depressive patients implies a link between inflammation, depression, and treatment. Further animal studies are needed to test antidepressive effects using antagonists of specific TLRs.
